# Modified Van Assche magnetic resonance imaging-based score for assessing the clinical status of anal fistulas

**DOI:** 10.1097/MD.0000000000020075

**Published:** 2020-05-08

**Authors:** Wei-Guo Wang, Wen-Zhu Lu, Chun-Mei Yang, Ke-Qiang Yu, Hong-Bo He

**Affiliations:** a Department of Integrated Traditional Chinese and Western Medicine, West China Hospital, Sichuan University; b Department of Integrated Traditional Chinese and Western Medicine, Cheng Du Shang Jin Nan Fu Hospital, Chengdu, Sichuan Province, China.

**Keywords:** anal fistula, anal fistula recurrence, magnetic resonance imaging, risk factors, Van Assche MRI-based score

## Abstract

The modified Van Assche magnetic resonance imaging (MRI)-based score is a feasible system to assess the clinical status of anal fistulas in Crohn disease. In this study, we evaluated this score's association with clinical status in patients with anal fistulas (AFs).

We included all patients with AF who underwent contrast-enhanced pelvic MRI and surgery between January 2011 and December 2016. The score was evaluated retrospectively preoperatively and 1, 3, and 6 months postoperatively. Univariate and multivariate analyses of the risk factors for AF recurrence were also performed.

We retrospectively analyzed data for 104 patients. Twelve (11.5%) patients developed AF recurrence. We classified patients’ preoperative clinical status into three grades: 52 (50.0%) grade A, 31 (29.8%) grade B, and 21 (20.2%) grade C. The preoperative MRI-based score was significantly correlated with patients’ preoperative clinical status grade (Pearson correlation: 0.547; *P* < .001). The 3 preoperative clinical status grades showed significant (*F* = 23.303, *P* < .001) tendencies for associations with lower respective MRI-based scores. The incidence of AF recurrence decreased with the MRI-based score to 1-month postoperatively, then gradually increased (*F* = 60.863, *P* = .000). Long duration of disease, prior interventions, and high MRI-based score were independent risk factors for AF recurrence.

The MRI-based score objectively assessed the clinical status and disease activity of patients with AFs, with a high score being associated with severe clinical status and long recovery time.

## 1 Introduction

Anal fistula (AF) is a highly prevalent disease of the anorectum, with up to 10 incidences per 100,000 population per year and a male to female ratio of 1.8:1.^[1]^ AF commonly originates secondary to cryptoglandular infection and subsequent perianal abscess. After the abscess is drained, the remnant cavity becomes fibrotic and forms an anal fistulous tract that connects the internal opening (the cryptoglandular infection) with the external opening (the abscess drainage site).^[2]^

In addition to high prevalence, high recurrence is another feature of AFs, with rates as high as 10%.^[3]^ Because AF is often recurrent and involves:

i) the external opening sealing over,
ii) pus accumulation, and
iii) the abscess once again extending to the surface, the cessation of abscess drainage can be confusing, and the relief of clinical symptoms (e.g., suppuration, pruritus ani, fecal discharge, swelling, bleeding, fever, gross fluctuance, and abscess) can be deceptive. Accordingly, recurrence can severely affect a patient's quality of life.^[4]^

AFs are classified as “simple” or “complex” by the American Society of Colon and Rectal Surgeons.^[5]^ Considering the chronic inflammation of the etiology, surgery is the mainstay of therapy. The courses and directions of simple AFs can easily be explored; however, it may be difficult to explore complex fistulas during surgery. Recurrence always develops at the residual AF tract following incomplete resection; therefore, it is crucial to perform imaging examinations to detect the invisible internal details of an AF tract before surgery, after treatment, and during follow-up. Currently, the most popular noninvasive imaging examinations include transrectal ultrasonography, magnetic resonance imaging (MRI), and computed tomography.^[6]^ Among these methods, MRI has the advantages of providing good discrimination of different soft tissue types, a wide field of view, multiplanar image acquisition, and no ionizing radiation hazard.^[7]^ The value of pelvic MRI to reveal the anatomical relationship of the fistula with the levator ani and the ischioanal/ischiorectal fossa has been demonstrated in previous studies.^[8,9]^

A simple and feasible MRI-parameter-based score was proposed by Van Assche in 2003 to assess the clinical behavior of anal fistulizing Crohn disease before and after treatment with infliximab remission induction therapy.^[7]^ A high MRI-based score was confirmed to be associated with more severe clinical behavior.^[10]^ In 2011, Horsthuis et al added T1 hyperintensity and infiltrate to the score to form the modified Van Assche MRI-based score to facilitate more detailed classification of inflammatory conditions (Table 1).^[11]^ This modified MRI-based score was reported to provide accurate assessment of the invisible internal details of an AF tract.^[7,11]^ Similar invisible detailed internal characteristics of chronic fistula develop in AF patients without anal fistulizing Crohn disease. Dirk et al reported that the modified Van Assche MRI-based score could be used for AFs not related to Crohn disease in their 2014 review describing MRI of AFs.^[12]^ However, to date, few studies have evaluated extended applications of the Van Assche MRI-based score, and its precise role in assessing the clinical status of AFs remains unclear. Therefore, we retrospectively evaluated the association between the Van Assche MRI-based score and the clinical status of patients who presented with AF over a 5-year period at West China Hospital and the value of the score to access AF recurrence after therapy.

**Table 1:**
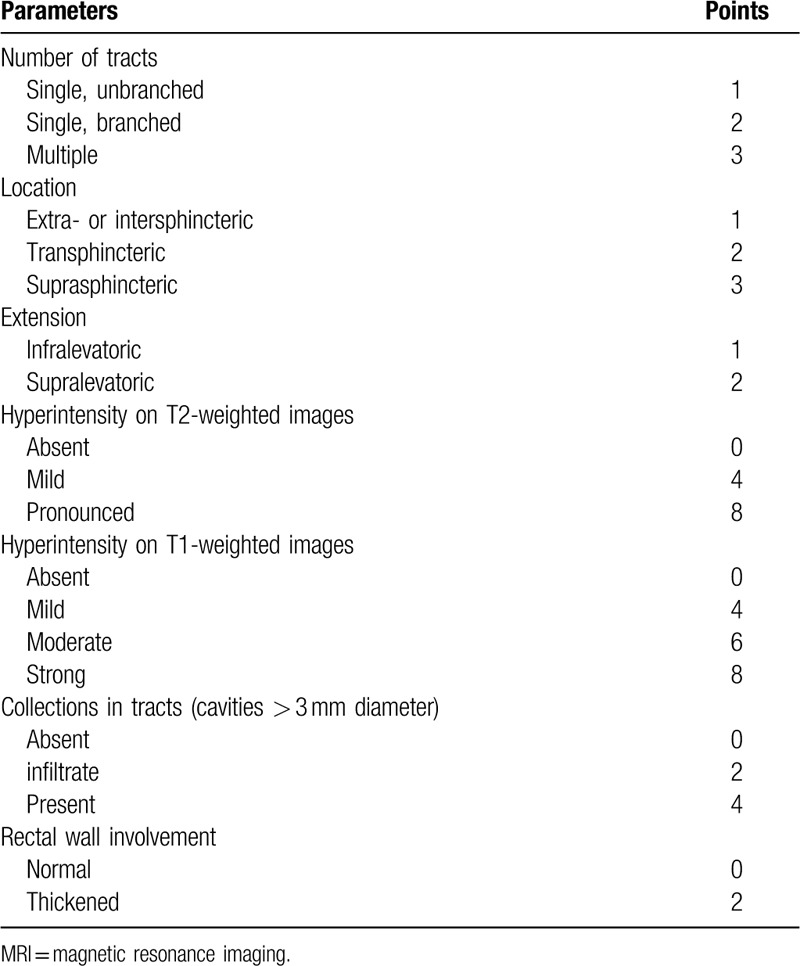
Modified Van Assche MRI-based score.

## 2 Materials and methods

### 2.1 Patients’ characteristics

We retrospectively evaluated data for 117 patients with AF who underwent contrast-enhanced pelvic MRI and surgery at West China Hospital (Sichuan, China) between January 2011 and December 2016, after the approval by the Ethics committee of West China Hospital. Patients comprised 76 men (65.0%) and 41 women (35.0%). We excluded patients with inflammatory bowel disease, traumatic injury, tuberculosis, pregnancy, or radiation-induced AF.

Patients’ electronic medical records were thoroughly reviewed, and data for the following basic characteristics were collected: age, sex, smoking, alcohol use, body mass index, duration of disease, prior interventions for AFs, Charlson Comorbidity index, surgery type (fistulectomy, fistulotomy, cutting seton), and healing time. Patients were followed for 1 to 6 months postoperatively (range: 32–191 days). Thirteen patients were lost during follow-up, leaving 104 patients for the final retrospective analysis.

### 2.2 Perioperative preparation and surgical technique

All patients underwent full bowel preparation with oral lavage solution before surgery, but did not receive preoperative antibiotics. Postoperatively, a liquid diet was required for 48 hours, bulking laxative was prescribed for 1 week, and we required that patients clean the anal wound after defecation for 2 weeks. To detect evidence of residual or recurrent AF, patients underwent a clinical follow-up each week for 1 month postoperatively, and were then followed 3 and 6 months postoperatively.

During surgery, we induced general anesthesia and placed patients in the dorsal lithotomy position. Inspection, palpation, digital rectal examination, and anoscopy were conventional steps at the beginning of surgery. We used a fistula probe to identify the AF tract and injected methylene blue into the external opening to confirm the presence of dye at the internal opening, as necessary. The decision to use fistulectomy, fistulotomy, and/or cutting seton was made preoperatively according to the anatomy of the fistula and the relationship of the tract with the sphincter mechanism. In patients with high fistulas, fistulectomy was performed to completely excise the AF tract. In patients with a large cavity in the AF tract, fistulotomy was performed to open the AF tract and to assist drainage of the cavity. In patients with complex transsphincteric fistulas, the portion of the AF tract outside the sphincter complex and granulation tissue was opened, and the portion of the AF tract inside the sphincter complex was looped with a tight seton, which gradually cut through the sphincter muscle and healed over time. The seton was left in place for at least 2 weeks, when it usually fell off.

### 2.3 MRI-based score and classification of the AF clinical status

The MRI-based scores were retrospectively assessed from imaging data retrieved from the electronic database for preoperative, and 1-, 3-, and 6-month postoperative data. Of the 104 patients, MRI results were available in the following proportions: preoperative: 93.5%; 1 month postoperative: 91.2%; 3 months postoperative: 76.8%; and 6 months postoperative: 79.6%.

We used the criteria of the modified Van Assche MRI-based score in this study **(**Table 1),^[7,11]^ which includes both anatomical parameters (number of tracts, location, and extension) and inflammatory parameters (hyperintensity on T2-weighted [T2W] images, hyperintensity on T1-weighted [T1W] images, collections in the tracts, and rectal wall involvement). The final score ranges from 0 to 30, depending on the weight of each parameter, with a high score indicating severe AF status.

We evaluated patients’ clinical status using the clinical status score classification,^[13]^ which includes the following 5 symptoms/signs: discharge, pain or discomfort, number of tracts, activity restrictions, and induration. Each parameter has a value of 1 to 3, and the patient's clinical status is classified into 1 of 3 grades according to the total score: grade A: 5–6 points; grade B: 7–9 points; and grade C: 10–15 points (Table 2). A high grade indicates AF with a severe clinical status.

**Table 2:**
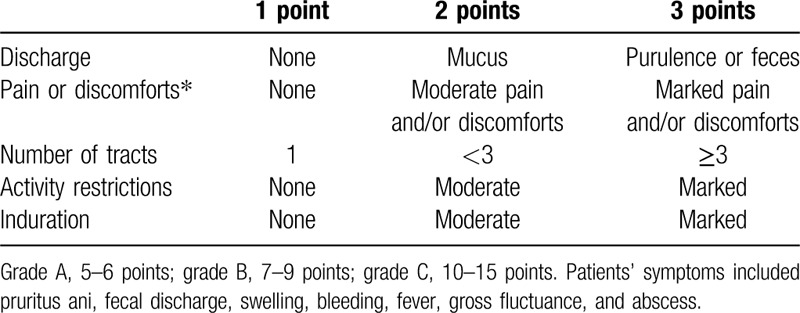
Clinical status classification.

### 2.4 Imaging technique

T1W two-dimensional (2D) turbo spin-echo (TSE) sequences (repetition time [TR]: 500 ms; echo time [TE]: 10 ms; matrix: 270 × 270; slice thickness: 4 mm; transverse planes), T2W 2D TSE sequences (TR: 3000 ms; TE: 500 ms; matrix: 350 × 270; slice thickness: 4 mm; transverse, sagittal, and coronal planes), and a fat-suppressed T1W 2D TSE sequence (TR: 450 ms; TE: 10 ms; matrix: 270 × 270; slice thickness: 4 mm; transverse planes) were obtained, in accordance with the standardized protocol for perianal disease. Bowel preparation was not performed before MRI examination, and contrast agent was not injected into the cavity of the AF during the MRI examination. Patients were placed in the supine position with the coil centered on the pelvis. Contrast-enhanced pelvic MRI was performed on a 3.0-T scanner (Philips Medical Systems, Best, the Netherlands) with an 8-channel phased array surface coil. The radiologist was blinded to patients’ clinical information during the MRI evaluation.

### 2.5 Statistical analysis

Normally distributed variables are reported as mean and standard deviation and were compared using *t* tests. Non-normally distributed variables are expressed as median (range) and were compared using Mann–Whitney U-tests. Categorical data were compared using chi-squared tests with Yates continuity correction in a two-way contingency table, or Fisher exact test. Univariate and multivariate analyses of risk factors for AF were performed to adjust for confounding factors. The univariate analysis included all the potential factors that had been retrospectively collected, while the multivariate analysis included the potential factors with a *P* value ≤ .05 in the univariate analysis, and we used a binary logistic regression model with conditional backward selection of potential factors. The results of the multivariate logistic regression analysis are expressed using *P* values, odds ratios (ORs), and 95% confidence intervals (CIs). The courses of the MRI-based scores preoperatively, and 1, 3, and 6 months postoperatively were analyzed using two-way repeated measures analysis of variance. All statistical analyses were performed using IBM SPSS version 22 (IBM Corp., Armonk, NY), with significance defined as *P* ≤ .05.

## 3 Results

### 3.1 Patients’ basic characteristics

Patients’ characteristics are shown in Table 3. The preoperative conditions included the following: 37.5% (39/104) abused alcohol, 37.5% (39/104) had coexisting diseases, and 12.5% (13/104) had undergone prior AF surgery. The mean Charlson Comorbidity index was 0.58 ± 0.90 (range: 0–5), and the mean duration of disease was 1.51 ± 2.41 years (range: 0.1–12.9 years). The surgical procedures performed included 26 (25.0%) fistulectomies; 29 (27.9%) fistulotomies; and 49 (47.1%) cutting setons. A total of 104 patients with a mean age of 43.2 ± 17.9 years (range: 18–71 years) eventually healed, with a mean healing time of 1.37 ± 2.09 months (range: 2–106 days). However, 12 (11.5%) patients whose clinical symptoms did not improve developed AF recurrence.

**Table 3:**
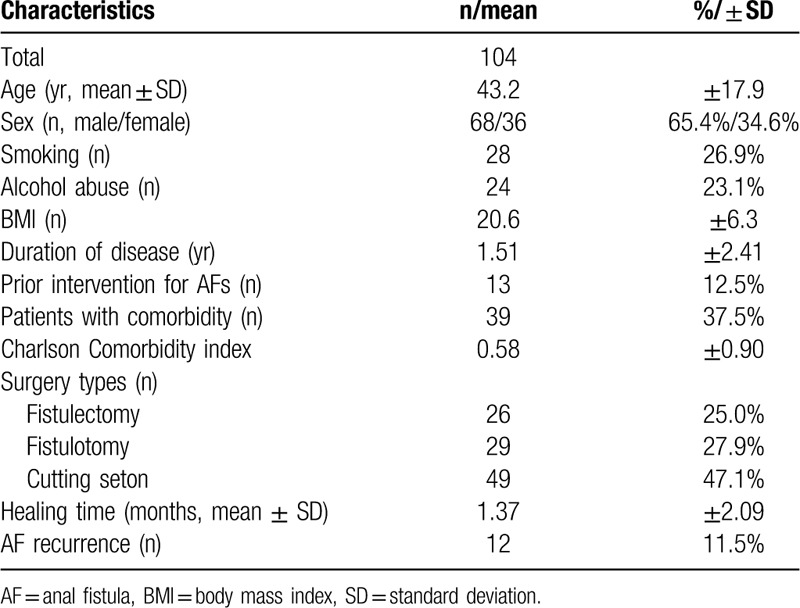
Patients’ basic characteristics.

### 3.2 Preoperative clinical status score and MRI-based score

Patients’ clinical status scores and MRI-based scores were retrospectively evaluated regarding the preoperative score. The mean preoperative clinical status score was 7.78 ± 2.99 (range: 5–15), with patients graded in order of increasing severity as: 50.0% (52/104) grade A, 29.8% (31/104) grade B, and 20.2% (21/104) grade C. Patients’ preoperative MRI-based scores ranged from 11 to 28 points, with a mean of 17.26 ± 4.40 points. The correlation between patients’ preoperative clinical status scores and their MRI-based scores was statistically significant (Pearson correlation = 0.547, *P* < .001; Table 4).

**Table 4:**
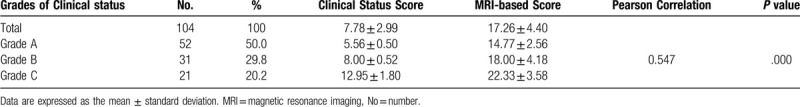
Preoperative MRI-based scores for the different clinical status grades.

### 3.3 The course of the MRI-based scores

**Figure 1:**
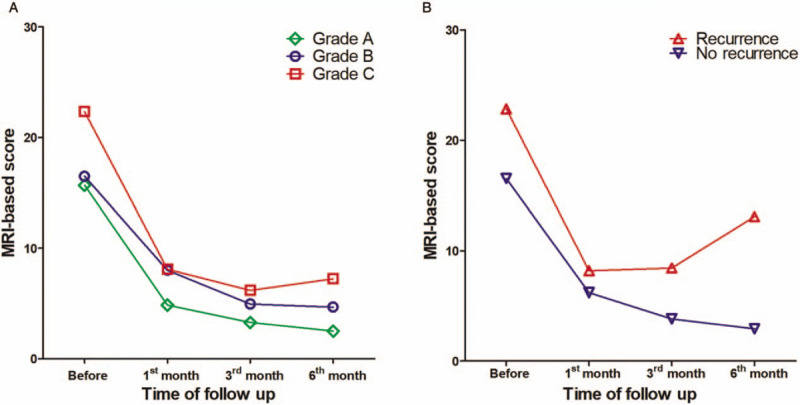
The course of the MRI-based score. The modified Van Assche MRI-based score was assessed preoperatively, and 1, 3, and 6 months postoperatively. A, shows the courses for the three preoperative clinical status grades. The total score decreased immediately when comparing preoperative and 1-month postoperative scores, and then changed to a moderate decreasing trend to 6 months post-surgery. The courses of the scores for the three grades showed a decrease from grade C to grade B to grade A over time, with the grade C group scores decreasing from a preoperative mean score of 22.33 ± 3.58 to 3.74 ± 5.84 6 months postoperatively; grade B scores decreasing from 18.00 ± 4.18 to 2.13 ± 3.27; and grade A scores decreasing from 14.77 ± 2.56 to 1.75 ± 2.25 (*F* = 23.303, *P *= .000). B, shows a comparison of the courses of the scores regarding the presence or absence of AF recurrence. The scores for the no-recurrence group decreased gradually, while the scores for the recurrence group decreased to a low point 1 month postoperatively, and then increased gradually to 6 months postoperatively; the courses of the scores differed significantly between the two groups (*F* = 60.863, *P* = .000). Data are expressed as the mean of the MRI-based scores. AF = anal fistula; MRI = magnetic resonance imaging.

We analyzed patients’ MRI-based scores preoperatively, and 1, 3, and 6 months postoperatively. Figure 1A shows the courses of the MRI-based scores, compared in pairs between the 3 preoperative clinical status grades. The total course showed a time trend for a gradual decrease in MRI-based scores. The scores decreased noticeably from before surgery to 1 month postoperatively, and then changed to a moderate decreasing course from 1 to 6 months postoperatively. The three preoperative clinical status grades showed significant tendencies to decrease with the MRI-based score, with the grade C group showing a decrease 6 months postoperatively from a preoperative mean of 22.33 ± 3.58 to 3.74 ± 5.84, grade B decreasing from 18.00 ± 4.18 to 2.13 ± 3.27, and grade A from 14.77 ± 2.56 to 1.75 ± 2.25 (Table 5). Furthermore, the courses of the grades differed significantly different between the three groups (*F *= 23.303, *P <* .001).

**Table 5:**
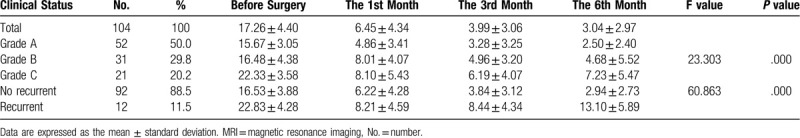
MRI-based scores for the different clinical status grades preoperatively and 1, 3, and 6 month postoperatively.

Figure 1B shows the courses of the MRI-based scores for patients with or without AF recurrence. The MRI-based score in patients without AF recurrence decreased gradually from a preoperative mean of 16.53 ± 3.88 to 2.94 ± 2.73 6 months postoperatively. However, patients with AF recurrence showed an initial rapid fall in the MRI-based score from a preoperative mean of 22.83 ± 4.28 to 8.21 ± 4.59 1 month postoperatively, with the score then gradually increasing to a mean of 13.10 ± 5.89 6 months postoperatively. The courses of the MRI-based scores differed significantly between the patients with and without AF recurrence (*F* = 60.863, *P <* .001; Table 5).

### 3.4 Risk factors for AF recurrence

Univariate and multivariate analyses were performed to identify the risk factors for AF recurrence (Table 6). The univariate analysis showed that AF recurrence was significantly associated with a long duration of disease (3.86 ± 2.17 years vs 1.36 ± 1.49 years, long duration vs short duration, respectively; *P *< .001), prior interventions (66.7% [8/12] vs 20.7% [19/92], AF recurrence vs no recurrence, respectively; *P <* .001), high number of comorbidities (75.0% [9/12] vs 32.6% [30/92], high number vs low number, respectively; *P *= .004), high Charlson Comorbidity index (1.17 ± 0.94 vs 0.50 ± 0.87, high vs low, respectively; *P *= .015), and high MRI-based score (22.83 ± 4.28 vs 16.53 ± 3.88, high score vs low score, respectively; *P <* .001). The multivariate analysis showed that the independent risk factors for AF recurrence were long duration of disease (*P *= .021, OR = 1.581, 95% CI: 1.080–2.336), prior interventions (*P *= .041, OR = 6.216, 95% CI: 1.080–35.780), and high MRI-based score (*P *= .001, OR = 1.386, 95% CI: 1.142–1.682).

**Table 6:**
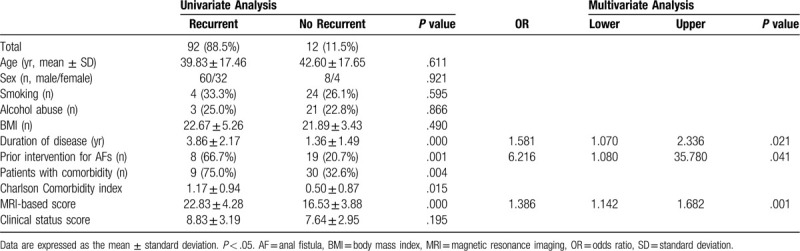
Analysis of the risk factors for AF recurrence.

## 4 Discussion

Our results in this study showed that patients’ preoperative MRI-based scores correlated significantly with their clinical status scores. Savoye-Collet et al evaluated the MRIs of 20 patients to assess perianal fistulas and correlate the changes in the MRI-based score with clinical outcomes .^[14]^ The authors found a significant improvement in the MRI-based scores, particularly regarding T2 hyperintensity, in patients achieving a good therapeutic effect.^[14]^ Studies have also demonstrated that the MRI-based parameters are sufficient to delineate the location of the AF tracts^[15]^ and their inflammatory extent.^[16]^ Spencer et al performed a pilot analysis evaluating the clinical course and therapeutic response of AFs,^[17]^ and concluded that preoperative contrast-enhanced T1W and axial T2W sequences provided adequate information to guide surgery. Additionally, fat-suppressed T1-weighted images have been shown to be useful for detecting tracts that are less conspicuous on T2-weighted images.^[2,18]^ Furthermore, the inflammatory criteria of the MRI-based score improved more consistently than the anatomical criteria.^[7]^ Furthermore, some studies claimed good interobserver reproducibility and good consistency amongst the parameters in the MRI-based scoring system.^[7,11,19]^ These results demonstrated that preoperative MRI-based scores can be used to evaluate patients’ clinical status. For surgeons, combining the MRI-based score and patients’ clinical status grade may be useful to accurately evaluate a patient's condition; however, the low number of cases limits the reliability of existing findings.^[11]^ Therefore, large sample trials should be considered to further evaluate the value of each MRI-based parameter in the score, and to further modify the MRI-based score, if necessary.

Despite the fact that surgery opens the external opening of the AF and helps drain the abscess, AF tracts persist with varying degrees of residual inflammation, indicating that persistent inflammation influences cicatrization of the AF tract.^[7]^ In the long follow-up (6 months) of the course of the MRI-based score after surgery, we found a gradually decreasing score with improved patients’ clinical status, indicating that a decreasing MRI-based score may reflect healing of the invisible internal AF tract. MRI is valuable for the assessment and follow-up of disease activity and the severity of inflammatory bowel disease,^[20]^ and further developments in MRI, including diffusion-weighted sequences and magnetization transfer ratio, are expected to assist in characterizing patients’ clinical status.^[21,22]^

Some studies have reported a declining course in MRI-based scores with treatment and remission of anal fistulizing Crohn’ disease.^[7,23]^ Furthermore, we found that patients with AF recurrence showed an initial decreasing MRI-based score, but that this reversed to an increase after 1 month postoperatively. This finding is consistent with the features of AF recurrence, which usually develops 1 to 3 months postoperatively, and relapsed inflammation develops inside the AF tract. For surgeons, these results confirm the value of this scoring system for assessing changes in a patient's condition, and for following AF patients to detect recurrence.

The univariate and multivariate analyses of risk factors in this study showed that long duration of disease, prior interventions, and high MRI-based scores were independent predictors of AF recurrence. AF recurrence often involves a long history of recurrent distressing clinical symptoms. Most patients with recurrent AF have a long disease duration and have undergone multiple prior interventions.^[24]^ For surgeons, vigilance is required, and outcomes should be monitored closely in patients with a long duration of AF and a history of prior interventions.^[25]^ When considering the MRI-based score as an independent predictor, the following 3 measures should be taken to reduce and/or minimize the incidence and severity of AF recurrence: First, patients with a high preoperative MRI-based score should be monitored before surgery, especially those with perianal inflammation. Second, measures to relieve perianal inflammation and to drain collections in the AF tracts (e.g., incising the external opening, drainage seton, and hip bath) should be considered before surgery.^[26]^ Third, the MRI-based score should be used to follow patients with AF and to evaluate patients with risk factors for AF recurrence.^[12]^

Despite the positive findings, our study has the following limitations: First, this was a retrospective study affected by patients lost to follow-up, selection bias, and information bias, which limits the reliability of our results to assess the severity of the clinical status of patients with AF. Second, the relatively small number of included patients prevented us from evaluating the efficacy of each parameter in the modified Van Assche MRI-based score for AF. Data for each separate parameter should be collected in a large-scale prospective study to evaluate the full extent of the clinical applicability of the Van Assche MRI-based score.

## 5 Conclusion

The modified Van Assche MRI-based score objectively indicates the clinical status and disease activity of AFs, and a change in the MRI-based score reflects the course of the clinical status of AF. A high score is associated with severe clinical status and long recovery time, and long disease duration, prior interventions, and high MRI-based scores are independent predictors of AF recurrence. The modified Van Assche MRI-based score is a potentially useful indicator to assess patients’ clinical status before surgery, to follow patients with AF, and to evaluate treatment efficacy.

## Acknowledgments

We thank Karl Embleton, PhD, and Jane Charbonneau, DVM, from Liwen Bianji, Edanz Group China (www.liwenbianji.cn/ac), for editing the English text of a draft of this manuscript.

## Author contributions

**Conceptualization:** Hong-Bo He.

**Formal analysis:** Wei-Guo Wang.

**Investigation:** Wen-Zhu Lu.

**Methodology:** Chun-Mei Yang.

**Resources:** Ke-Qiang Yu.

**Software:** Chun-Mei Yang.

**Supervision:** Ke-Qiang Yu.

**Validation:** Chun-Mei Yang.

**Visualization:** Wen-Zhu Lu.

**Writing – original draft:** Wei-Guo Wang.

**Writing – review & editing:** Hong-Bo He.
